# Spatial distribution and predictors of domestic violence against women: evidence from analysis of Ethiopian demographic health survey 2016

**DOI:** 10.1186/s12905-021-01465-4

**Published:** 2021-09-15

**Authors:** Elias Seid, Tesfahun Melese, Kassahun Alemu

**Affiliations:** 1 Jimma University Medical Center, 378, Jimma, Ethiopia; 2 Department of Health Informatics, Institute of Public Health, University of Gondar, 196, Gondar, Ethiopia; 3 Department of Epidemiology and Biostatistics, Institute of Public Health, University of Gondar, 196, Gondar, Ethiopia

**Keywords:** Domestic violence, Spatial distribution, Ethiopia

## Abstract

**Background:**

Violence against women particularly that is committed by an intimate partner is becoming a social and public health problem across the world. Studies show that the spatial variation in the distribution of domestic violence was commonly attributed to neighborhood-level predictors. Despite the prominent benefits of spatial techniques, research findings are limited. Therefore, the current study intends to determine the spatial distribution and predictors of domestic violence among women aged 15–49 in Ethiopia.

**Methods:**

Data from the Ethiopian demographic health survey 2016 were used to determine the spatial distribution of domestic violence in Ethiopia. Spatial auto-correlation statistics (both Global and Local Moran’s I) were used to assess the spatial distribution of domestic violence cases in Ethiopia. Spatial locations of significant clusters were identified by using Kuldorff’s Sat Scan version 9.4 software. Finally, binary logistic regression and a generalized linear mixed model were fitted to identify predictors of domestic violence.

**Result:**

The study found that spatial clustering of domestic violence cases in Ethiopia with Moran’s I value of 0.26, Z score of 8.26, and *P* value < 0.01. The Sat Scan analysis identifies the primary most likely cluster in Oromia, SNNP regions, and secondary cluster in the Amhara region. The output from regression analysis identifies low economic status, partner alcohol use, witnessing family violence, marital controlling behaviors, and community acceptance of wife-beating as significant predictors of domestic violence.

**Conclusion:**

There is spatial clustering of IPV cases in Ethiopia. The output from regression analysis shows that individual, relationship, and community-level predictors were strongly associated with IPV. Based upon our findings, we give the following recommendation: The government should give prior concern for controlling factors such as high alcohol consumption, improper parenting, and community norm that encourage IPV that were responsible for IPV in the identified hot spot areas.

## Background

The term domestic violence refers mainly to intimate partner violence, but may also include abuse by any member of a household. In recent years, violence against women, particularly intimate partner violence, has become a social and public health issue throughout the world. According to the 2017 WHO report, one in three women (35%) globally has been a victim of domestic violence [1]. Although the burden of the problem varies from country to country, available studies show that the burden of the problem is high in African countries [2–6].

In Ethiopia, violence against women and girls continues to be a major problem. According to EDHS 2016 report, 34% of ever-married women age 15–49 have experienced either physical, sexual, or emotional spousal violence [7]. Likewise, a systematic review of 15 articles on domestic violence from 2000 to 2014 also shows that the lifetime prevalence of domestic violence against women by an intimate partner was ranged from 20 to 78% [8].

Spatial statistical techniques give a prominent benefit for examining the distribution of health problems. Research evidence shows that the distribution of IPV spatially varies. This variation was attributed to community-level characteristics. High prevalence was observed in areas where black American women reside in the USA [9] and, around entertainment areas in Canada [10]. Likewise, clusters of IPV have also been observed in poorly educated areas, high public disorder, and high concentrations of immigrants [11].

Despite the benefits of spatial techniques, it has not been well used in health literature. In the Ethiopian context, the spatial distribution pattern of IPV has not been broadly explored. As far as my literature research is concerned, no article has been found which shows the distribution of domestic violence in Ethiopia using this technique. Therefore, the current study uses spatial analysis tools to determine the distribution and factors associated with IPV among women aged 15–49 in Ethiopia.

## Methods

### Study design and setting

The study uses data that was extracted from EDHS 2016 dataset. In EDHS 2016, a community-based cross-sectional study was conducted by the Central Statistical Agency (CSA) from January 18 to June 27, 2016, in Ethiopia.

**Fig. 1:**
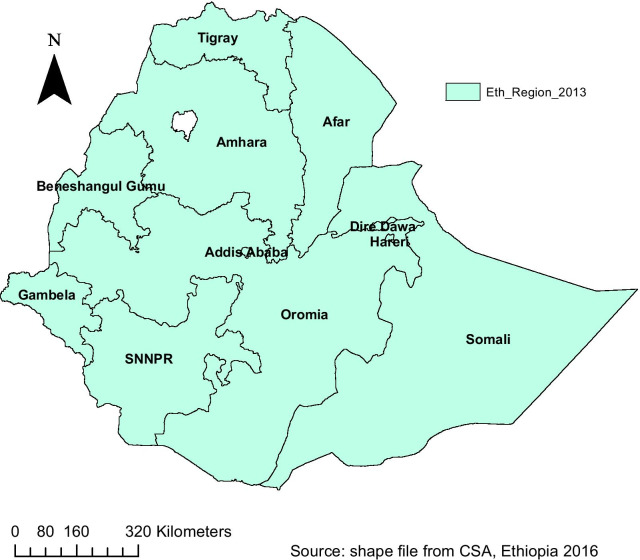
Map of Ethiopia where the study is undertaken. Shapefile from Ethiopia Central Statistical Agency, 2013

Ethiopia is an ancient country located in the Horn of Africa from 3^0^ to 14^0^ N and 33^0^ to 48^0^E. Ethiopia has a total area of 1,100,000 square kilometers and with over 110 million population and this makes it the second populous country in Africa. Ethiopia is a nation with around 80 ethnic groups. From this, the four largest are Oromo, Amhara, Somali, and Tigrayans. Regarding climatic conditions, Ethiopia is an ecologically diverse country ranging from desert in the eastern part to tropical rainforest to south and south-west. Figure 1 shows map of Ethiopia where the study has been conducted.

### Sample size and sampling technique

EDHS 2016 used two stages stratified cluster sampling technique where each region was stratified into urban and rural areas, yielding 21 sampling strata. In the first stage, a total of 645 Enumeration Areas (EAs) were selected with probability proportional to EA size and with independent selection in each sampling stratum [7]. In the second stage, a fixed number of 28 households per cluster were selected and only one woman per household was randomly selected for interview. Finally, a total of 5860 women aged 15–49 were asked questions about domestic violence against women. All women aged 15–49 and who are the usual members of selected households and who stayed in the household the night before the survey were eligible to be interviewed [7].

### Data collection procedures

In the 2016 EDHS, questionnaires from the standard Demographic and Health Survey were adapted to reflect the population and health issues relevant to Ethiopia. The questionnaire was translated into local languages (Amharic, Tigrigna, and Oromiffa) to appropriately collect the information needed.

Information about IPV was obtained by asking ever married woman a 13 item question; of which, 7 measures physical IPV, 3 emotional IPV, and 3 sexual IPV. Table 1 contains questions used to measure emotional, physical, and sexual violence committed by their partners.

**Table 1:** Questions used to measure intimate partner violence against women

Questions	IPV type
Did your partner ever push you, shake you, or throw something at you?	Physical IPV
Did your partner ever slap you?	
Did your partner ever twist your arm or pull your hair?	
Did your partner ever punch you with his/her fist or with something that could hurt you?	
Did your partner ever kick you, drag you, or beat you up?	
Did your partner ever try to choke you or burn you on purpose?	
Did your partner ever threaten or attack you with a knife, gun, or any other weapon?	
Did your partner ever physically force you to have sexual intercourse with him even when you did not want to?	Sexual IPV
Did your partner ever physically force you to perform any other sexual acts you did not want to?	
Did your partner ever force you with threats or in any other way to perform sexual acts you did not want to?	
Did your partner ever say or do something to humiliate you in front of others?	Emotional IPV
Did your partner ever threaten to hurt or harm you or someone close to you?	
Did your partner ever insult you or make you feel bad about yourself?	

### Variables of the study

The dependent variable of the study is the experience of domestic violence by women aged 15–49. A woman is said to be experienced domestic violence if she ever faced either emotional, physical, or sexual violence or a combination of the three committed by her partner.

The independent variables are categorized into individual-level, household/relationship level, and community-level factors. Table 2 lists variables used in the study with their measurements.

**Table 2:** List of variables used in the study with their measurement description

Level	Variables	Measurement
Individual-level	Age	The age of women categorized as 15–19, 20–24, 25–29, 30–34, 35–39, 40–44, 45–49
	Educational status	Maximum educational level categorized as uneducated, primary, secondary, and above
	Religion	Respondents religion was categorized as Orthodox, Muslim, protestant, catholic, traditional, and others
	Partner drink alcohol	Classified as ‘yes’ if partner drinks alcohol and ‘no’ otherwise
	Witnessing family violence	‘Yes’ or ‘no’ based on their answer to the question “as far as you know, your father ever hit your mother?”
	Attitude on IPV	Women were asked questions about whether the husband is justified for hitting his wife for the following woman actions: if she goes out without telling him; neglects their children; argues with him; refuses to have sex with him, and burns the food. If they answer ‘yes’ to either of the above questions they were considered as ‘accept IPV’ and ‘don’t accept IPV’ otherwise
Household/relationship level	Number of children	Grouped as no child, 1–3 children, 4–6 children and above
	Household wealth index	Measured based on the number and kind of goods households have and housing characteristics (drinking water, toilet facility, flooring material, and availability of electricity). This was generated using Principal component analysis and classified into quintiles from 1 = very poor to 5 = very rich
	Decision-making power	Labeled ‘yes’ if a woman was involved in all decisions regarding her own healthcare, major household purchases, and visits to her family or relatives
	Marital controlling behaviors	a woman asked if her partner demonstrates one of the following controlling behaviors: he is jealous or angry if she talks to other men, frequently accused her of being unfaithful, do not permit her to meet her female friends, tries to limit her contact with her family, and insists to know where she is at all times. She is considered as in controlled condition if she answers yes to either of the above questions and otherwise not
Community-level	Community’s IPV acceptance level	Categorized as ‘low’ if the proportion of women who have IPV accepting attitude in the community was between 0–66.7percent and, as’ high’ if the proportion was greater than 66.8%
	Female literacy in the community	Categorized as low if the proportion of women who attended primary or secondary education was 0–36.4% and categorized as high if the proportion was 36.5–100%
	Place of residence	Urban or rural based on where the woman lives
	Region	Region of the woman where she is living

### Data processing and analysis

Statistical analysis of the data was performed on SPSS version 25. Cross-tabulations and summary statistics have been carried out to describe populations by age, level of education, place of residence, and region. Binary logistic regression and a two-level generalized linear mixed model were employed to identify predictors of domestic violence. Finally, a model comparison between the models was performed based upon the Log-likelihood ratio test to choose the best-fitted model.

### Spatial analysis of domestic violence

ArcGIS 10.7 software was used for spatial analysis of the data. Spatial autocorrelation (Global Moran’s I) statistics and Anselin local cluster analysis was performed to show the spatial distribution of domestic violence among woman aged 15–49 in Ethiopia. Global Moran’s I measure was used to verify whether domestic violence among women aged 15–49 is clustered, dispersed, or randomly distributed in Ethiopia.

Global Moran’s I calculates Moran's I Index value, Z score & *P* value. Moran’s I index close to -1 means domestic violence cases are dispersed whereas, close to 1 indicates that domestic violence cases are clustered. Statistically significant Z-score and *P* value ≤ 0.05 lead to rejection of the null hypothesis showing the existence of clusters of domestic violence. Statistically non-significant Moran’s I value (if *P* value > 0.05) will indicate domestic violence cases are randomly distributed throughout the country [12].

Anselin local Moran’s I was used to identify local level clusters of domestic violence. A positive Local Moran’s I indicate that the feature is surrounded by features with similar values and, such types of cases are called clusters. Whereas, a negative value for I indicates that the feature is surrounded by features with dissimilar values, and this was called an outlier [12].

Kuldorff’s Sat Scan version 9.4 software was used to identify the geographical locations of statistically significant clusters of domestic violence. Scan statistics use a scanning window that moves across the study area. Bernoulli's model was fitted to identify statistically significant locations of domestic violence clusters. The Bernoulli model was selected because the structure of the data shows the binomial [0/1] distribution. Women who have experienced domestic violence were considered as case and labeled1 whereas, those who do not experience as control and labeled 0. The default 50% of the population was used as an upper limit for cluster size; because it allows the detection of both small and large clusters of domestic violence. Statistically significant clusters were identified by *P* value and likelihood ratio tests.

### Multi-level logistic regression analysis

A two-level generalized linear mixed model was fitted by considering 4322 women aged 15–49 at level 1 nested within 645 clusters (communities) at level two. A multilevel analysis of the data takes three steps. The first step was fitting the null (intercept only) model without including predictor variables and the second step was a random intercept fixed coefficient model (model 2) by including individual and relationship level variables. The last was fitting a random intercept and fixed coefficient model (model 3) by incorporating community-level predictors.

### Model Comparison

Model comparison between the nested (null model and random intercept fixed coefficient model) and the logistic regression model was done to select the best-fitted model. The commonly used parameter for evaluation of model fitness is the Log-likelihood ratio test that compares the deviance (-log likelihood) of the models by subtracting the smaller deviance from the larger one. Deviance is an indicator that shows how well the model fits the data. A model with the lowest deviance is considered as the best-fitted model than with large deviance. In addition to the log-likelihood ratio test, Akaike’s information criterion (AIC) and Bayesian information criterion (BIC) were also used as measures of model fitness to select the best one. Similar to the log-likelihood ratio test, the model with a small AIC and BIC value is considered as the better model.

## Result

### Socio-demographic characteristics of respondents

A total of 3846 (weighted) women aged 15–49 were included for analysis. The majority 3219 (83.7%) of the respondents were from the rural part of the country and 1532 (39.8%) of them were from the Oromia region. The mean age of the respondents was 27.76 ± 9.1SD years and the majority, 2361 (61.1%) of the respondents do not attend formal education. Most of the respondents, 1526 (39.7.1%), were Orthodox by religion and 817 (21.2%) of them were from the richest family. Table 3 shows a cross-tabulation of the socio-demographic characteristics of the respondents with their experience of domestic violence.

**Table 3:** Cross-tabulation of socio-demographic characteristics of respondents with their experience of domestic violence

Socio-demographic characteristics	Domestic violence	
	No (%)	Yes (%)
Age in 5-year groups		
15–19	75.8	24.2
20–24	68.7	31.3
25–29	70.0	30.0
30–34	66.7	33.3
35–39	65.8	34.2
40–44	65.5	34.5
45–49	60.7	39.3
Education		
No education	65.2	34.8
Primary	68.0	32.0
Secondary	76.0	24.0
Higher	84.4	15.6
Residence		
Urban	78.6	21.4
Rural	65.4	34.6
Religion		
Orthodox	66.6	33.4
Catholic	75.9	24.1
Protestant	68.5	31.5
Muslim	69.3	30.7
Traditional	39.5	60.5
Other*	42.9	57.1
Wealth index		
Poorest	63.6	36.4
Poorer	65.1	34.9
Middle	63.1	36.9
Richer	67.5	32.5
Richest	77.8	22.2
Region		
Tigray	69.5	30.5
Afar	78.9	21.1
Amhara	65.3	34.7
Oromia	63.7	36.3
Somali	91.4	8.6
Benishangul Gumuz	67.5	32.5
SNNPR	71.0	29.0
Gambelia	63.6	36.4
Harari	62.5	37.5
Addis Ababa	77.2	22.8

*Any other type of religion the respondent follows

### Domestic violence

From women included in the analysis, 24% (95% CI 22.9%, 25.4%) of them have experienced emotional violence, 23.7% (95% CI 22.5%, 25%) physical violence and 10.1% (95% CI 9.3%, 11.1%) sexual violence by their intimate partner. In addition, 34% 95% CI (32.6%, 35.4%) women have experienced either type of violence by their intimate partner.

### Spatial distribution of domestic violence in Ethiopia

**Fig. 2:**
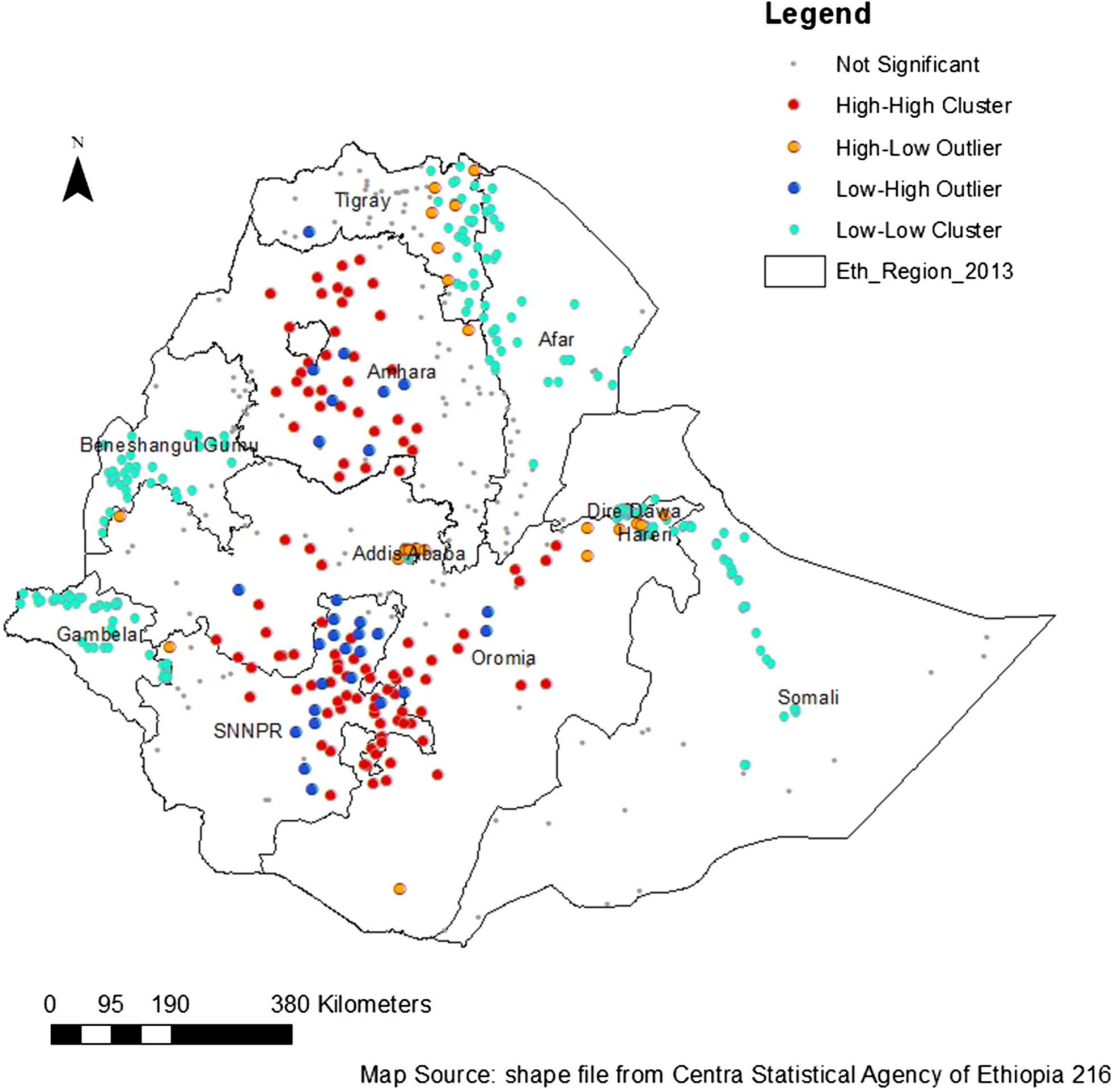
Output from Anselin Local cluster analysis of domestic violence in Ethiopia, 2016

The result of this study shows that the spatial distribution of domestic violence among women aged 15–49 in Ethiopia was non-random with Global Moran’s I 0.26 (*P* value < 0.01). The z score value of 8.29 indicating less than 1% likelihood that the observed clustering of domestic violence among women in Ethiopia is the result of random chance. The result from Anselin Local Moran’s I indicate the existence of hot spots, cold spots, and outlier clusters in the study area. Hot spot clusters are observed in Amhara regions (East Gojam and West Gojam zones), in the Oromia region (West Arsi, Guji, Bale, and Jimma zones), and in SNNP (Sidama, Gedio, Dawro, and Gamo Gofa zones). Cold spots have been observed in Benishangul Gumuz, Tigray (eastern, central, and southern areas), and the eastern part of the Somali region. Figure 2 shows Output from Anselin Local cluster analysis of domestic violence in Ethiopia.

### Sat scan analysis of domestic violence in Ethiopia

A total of 3 significant clusters was identified. Of these, one was considered the most likely (primary) cluster, while the remaining as a secondary cluster. The primary cluster was located in Oromia, Somalia, and some parts of SNNP regional states. In the region of Oromia, particularly in Guji, Borena, and Bale zones, in the region of Somalia, Liben, and Afder zones, and in the SNNP, Sidama zone were included. The primary cluster spatial window was centered at 5.203234 N, 40.0197322 E with18783 Km radius, LLRR of 39.55, and P—values < 0.0011. The relative risk tells us that women who live in this cluster have a 2.18 times higher risk of IPV than those who live outside the cluster. The *P* value is sufficient to reject the null hypothesis, indicating that this cluster is an actual and not randomly created cluster.

**Fig. 3:**
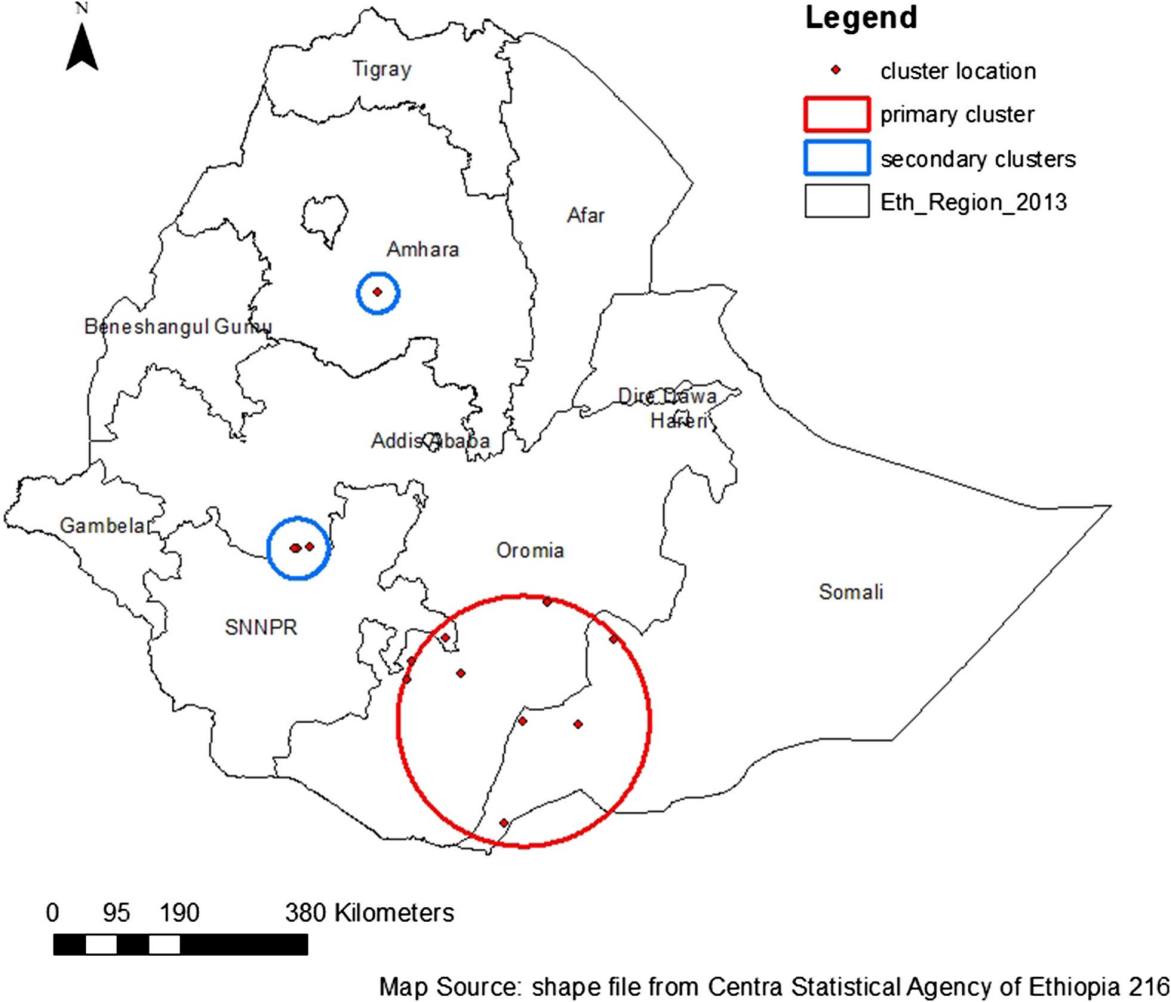
Output from Sat Scan analysis of domestic violence in Ethiopia, 2016

The secondary cluster was located in the Amhara region (in the Eastern Gojam zone) and, in the Oromia region (Jimma zone). The spatial window of secondary cluster detected by Sat Scan analysis centered at 10.984556 N, 38.044450 E with 29.42 km radius with a relative risk (RR) of 2.96 and log-likelihood ratio (LLR) of 28.56 with a *P* value of < 0.001. The bright red ring shown in Fig. 3 shows the primary significant cluster and the green ring shows the secondary cluster.

### Result of logistic regression

A binary logistic regression analysis was used to examine the association of predictive variables with domestic violence. According to the output from this, age, education, region, wealth index, partner’s education, partner alcohol use, respondent’s father ever beat mother, respondent afraid of her partner, marital controlling behaviors, and community acceptance of wife-beating shows a significant association with domestic violence.

The experience of domestic violence was increased with increasing in woman’s age. The Odds of experiencing domestic violence were 2 times higher for women aged 20–24 with (AOR = 2.09, 95% CI: 1.36, 3.17) and 3 times higher for women aged 45–49 with (AOR = 395% CI: 1.84, 5.15) when compared with those women aged 15–19.

Women from the richest family were 48% and those from richer families were 42% less likely to experience domestic violence when compared with those women from the poorest household with (AOR = 0.52, 95% CI 0.36, 0.75**)** and (AOR = 0.58, 95% CI 0.45, 0.77), respectively.

Partner education was also significantly associated with domestic violence. Women whose partner’s education is a secondary school were 42% and those with primary education were 19% less likely to experience domestic violence when compared to those with no education with (AOR = 0.58, 95% CI: 0.41, 0.82) and (AOR = 0.81, 95% CI: 0.66, 0.97), respectively.

Women whose partner drink alcohol were 2.6 times more likely to experience domestic violence when compared to those whose husband/partner does not drink alcohol (AOR = 2.62, 95% CI 2.09, 3.29)**.**

The Odds of experiencing domestic violence among women who witnessed family violence during childhood were 2.2 times higher than those who do not saw family violence (AOR = 2.24, 95% CI 1.81, 2.58).

Women whose partners exhibit at least one type of marital controlling behavior were 4.3 times more likely to experience domestic violence when compared to those whose who don’t exhibit any kind of marital controlling behavior with (AOR = 4.26, 95% CI: 3.55, 5.11).

The Odds of domestic violence was 4.4 times higher among women who were afraid of their partner most of the time and 2.3 times higher among those who were sometimes afraid of their partner when compared to those who don’t afraid of their partner (AOR = 4, 95% CI: 3.45, 5.61) and (AOR = 3.21, 95% CI 1.83, 2.81), respectively.

Women who live in communities where wife beating is highly acceptable were 1.4 times more likely to experience domestic violence when compared to those who live in communities where wife beating is less acceptable (AOR = 1.39, 95% CI: 1.16, 1.66). Table 4 displays the output from the binary logistic regression analysis.

**Table 4:** Output from the binary logistic regression analysis that shows factors associated with experiencing domestic violence among women age 15–49 in Ethiopia 2016

Variables		AOR	95% CI	
			Lower	Upper
Intercept	***	.024	.012	.050
Age				
45–49	***	3.08	1.84	5.15
40–44	***	2.59	1.56	4.29
35–39	***	2.50	1.56	4.01
30–34	***	2.35	1.49	3.73
25–29	***	2.03	1.31	3.14
20–24	***	2.08	1.36	3.17
15–19		1		
Highest education				
Higher		.76	.41	1.44
Secondary		1.07	.69	1.65
Primary	**	1.28	1.04	1.58
No education		1		
Religion				
Other		1.88	.84	4.19
Traditional	**	2.85	1.22	6.64
Muslim		1.28	1.00	1.63
Protestant		1.24	.93	1.65
Catholic		1.99	.86	4.58
Orthodox		1		
Wealth index				
Richest	***	.52	.36	.75
Richer	***	.58	.45	.77
Middle		.80	.62	1.03
Poorer		.78	.60	1.01
Poorest		1		
Number of living children				
> 6 children		1.01	.64	1.59
4–6 children		.98	.66	1.47
1–3 children		.99	.69	1.429
No child		1		
Husband/partner's education				
I don’t know		.79	.36	1.72
Higher		.78	.50	1.20
Secondary	***	.58	.41	.82
Primary	**	.81	.66	.97
No education		1		
Husband/partner drinks alcohol				
Yes	***	2.62	2.09	3.29
No		1		
Respondent's father ever beat her mother				
I don’t know		1.22	.85	1.75
Yes	***	2.16	1.81	2.58
No		1		
Respondent afraid of husband/partner				
Sometimes	***	2.26	1.83	2.81
Most of the time	***	4.41	3.45	5.61
Don’t afraid		1		
Number of unions				
More than once		1.13	.91	1.41
Once		1		
Type of place of residence				
Rural		.85	.57	1.26
Urban		1		
Marital controlling by partner				
I don’t know	***	11.16	4.96	25.06
Yes	***	4.26	3.55	5.11
No		1		
Attitude towards wife-beating				
Justified		1.11	.92	1.33
Not justified		1		
Decision-making power				
Yes		1.22	.94	1.58
No		1		
Community acceptance of WB				
High	***	1.39	1.16	1.66
Low		1		
Region				
Addis Ababa		1.08	.49	2.39
SNNP		.77	.51	1.18
Somali		.28	.06	1.16
Oromia		.95	.65	1.40
Amhara		1.13	.77	1.66
Afar		.00	.00	.00
Tigray		1		

***P value < 0.01; **P value < 0.05

### Result of multilevel logistic regression analysis

The null model is the first model in multilevel regression analysis in which only the intercept randomly varies across level two units without adjusting for predictor variables. The intercept-only model intends to verify the heterogeneity of communities experiencing domestic violence. The result from the null model shows that the variance of random factor is 0.716 with its calculated Z statistics of 7.35 and *P* value of 0.000. This shows that experiencing domestic violence among women aged 15–49 randomly varies across clusters. The ICC value shows that 21.4% of the variation in the outcome variable was explained by the grouping variable and the rest was by predictor variables.

The second model is a random intercept model that has a random intercept component and a fixed coefficient of individual and relationship level factors. The third model (full model) was developed by including community-level variables in model two. The output from this model shows that the experience of domestic violence was increased with an increase in women's age. The odds of experiencing domestic violence were 2.8 times higher among women whose age group was 30–34 and 4.2 times higher for those aged 45–49 when compared to women age 15–19 with (AOR = 2.8, 95% CI 1.05, 4.54) and (AOR = 4.2, 95% CI 1.82, 9.82), respectively.

Women from the richest family were 59% and those from richer families were 45% less likely to experience domestic violence when compared to those women from the poorest household with (AOR = 0.41, 95% CI 0.22, 0.77), (AOR = 0.55, 95% CI 0.36, 0.84), respectively.

Women whose partners drink alcohol were 2.7 times more likely to experience domestic violence when compared to those whose partners do not drink alcohol with (AOR = 2.7, 95% CI 1.84, 4.01).

The Odds of experiencing domestic violence among women who witnessed family violence during childhood were 2.5 times higher than those who do not saw family violence (AOR = 2.5, 95% CI 1.86, 3.37).

Women whose partners exhibit at least one type of marital controlling behavior were 4.2 times more likely to experience domestic violence when compared to those whose partners don’t exhibit any kind of marital controlling behavior with (AOR = 4.2, 95% CI 3.09, 5.63).

The Odds of domestic violence was 5.4 times higher among women who were afraid of their partner most of the time and 2.5 times higher among women who were sometimes afraid of their partner when compared to those who don’t afraid of their partner (AOR = 5.4, 95% CI 3.560, 8.132) and (AOR = 2.5, 95% CI 1.652, 3.726), respectively. Table 5 shows the output from multilevel logistic regression.

**Table 5:** Output from a random coefficient multilevel logistic regression that shows factors associated with experiencing domestic violence among women age 15–49 in Ethiopia 2016

Variables	Individual and relationship level (Model 2)				Full model (Model 3)			
		AOR		95% CI		AOR		95% CI
		Lower	Upper			Lower	Upper	
Intercept	***	.017	.006	.05	***	.03	.007	.10
Age								
45–49	***	4.35	1.87	10.08	***	4.22	1.82	9.82
40–44	***	3.89	1.50	10.08	***	3.82	1.45	10.04
35–39	**	3.04	1.25	7.40	**	2.95	1.21	7.18
30–34	**	2.78	1.18	6.53	**	2.74	1.16	6.45
25–29	**	2.26	1.06	4.81	**	2.24	1.05	4.79
20–24	**	2.19	1.05	4.55	**	2.18	1.05	4.54
15–19		1				1		
Highest education								
Higher	.63	.781	.28	2.17		.69	.240	2.001
Secondary	.82	.922	.44	1.91		.85	.404	1.824
Primary	.33	1.19	.82	1.73		1.19	.822	1.728
No education		1				1		
Religion								
Other		1.72	.22	13.37		1.86	.22	15.47
Traditional		3.82	.77	18.86		3.82	.71	20.33
Muslim		1.09	.69	1.72		1.17	.71	1.92
Protestant		1.05	.67	1.63		1.14	.68	1.91
Catholic		.82	.27	2.50		.88	.28	2.75
Orthodox		1				1		
Wealth index								
Richest	**	.493	.285	.854	***	.41	.22	.77
Richer	**	.563	.373	.851	***	.55	.36	.84
Middle		.799	.482	1.327		.78	.47	1.31
Poorer		.794	.549	1.147		.78	.54	1.13
Poorest		1				1		
Number of living children								
More than 6		.89	.42	1.88		.92	.44	1.94
4–6 children		.83	.42	1.62		.84	.43	1.63
1–3 children		1.02	.59	1.76		1.01	.58	1.74
No child		1				1		
Partner's education								
Higher		.68	.120	3.956		.66	.12	3.66
Secondary		.77	.37	1.60		.79	.38	1.64
Primary		.55	.29	1.02		.55	.29	1.04
No education		1				1		
Partner drinks alcohol								
Yes	***	2.75	1.88	4.02	***	2.71	1.84	4.01
No								
Respondent's father ever beat her mother								
I don’t know		1.35	.642	2.87		1.35	.641	2.87
Yes	***	2.49	1.86	3.34	***	2.51	1.86	3.37
No		1				1		
Respondent afraid of partner								
Sometimes	***	2.42	1.62	3.61	***	2.46	1.63	3.72
Most of the time	***	5.33	3.54	8.02	***	5.39	3.52	8.2
Don’t afraid		1				1		
Number of unions								
More than once		1.07	.74	1.55		1.05	.72	1.55
Once		1				1		
Women involved in decision making								
Yes		1.27	.741	2.19		1.27	.73	2.21
No								
The attitude of a woman to WB								
Justified		1.27	.953	1.69		1.24	.91	1.67
Not justified								
Marital controlling behaviors								
I don’t know	***	23.6	10.31	54.01	***	22.83	9.97	52.24
Yes	***	4.17	3.10	5.62	***	4.17	3.09	5.63
No		1				1		
Place of residence								
Rural							.57	.30
Urban						1		
The community acceptance level of WB								
High						1.30	.92	1.85
Low						1		
Region								
Addis Ababa						.84	.32	2.17
SNNP						.78	.42	1.47
Somali						.26	.06	1.16
Oromia						.93	.51	1.70
Amhara						1.04	.58	1.85
Afar						.00	00	.01
Tigray						1		

***P value < 0.01; **P value < 0.05

### Model comparison

The logistic regression model and the two-level generalized mixed model were compared based upon their log-likelihood ratio and the two criterion measures (AIC and BIC). The model with a small AIC and BIC measure was considered the best-fitted model.

The output from the analysis shows that employing a two-level generalized mixed model could not improve the model fitness. Rather logistic regression analysis is considered as the best-fitted model since it has significantly lower AIC and BIC values. Table 6 shows AIC and BIC values for logistic regression and generalized mixed model.

**Table 6:** Model comparison parameters of conventional logistic regression and multilevel logistic regression

	Logistic regression	Null model	GLM full model
-Log-Likelihood	1813.74	2277	1850
Akaike's Information Criterion (AIC)	3709.49	4560	3808
Bayesian Information Criterion (BIC)	3963.22	4572	4148

## Discussion

A nationally representative sample of EDHS 2016 data was used to determine the spatial distribution and determinants of spousal violence in Ethiopia. Almost one-third (34%) of women aged 15–49 have experienced domestic violence in their lifetime. And 24%, 23.5%, and 10.1% of women were emotionally, physically, and sexually abused by their partners, respectively. This finding is in line with WHO prevalence estimates of intimate partner violence for African countries [6], a study conducted in Ghana [2], and almost similar to the 2016 DHS national report [7]. This high prevalence suggests that domestic violence continues to be the main social and public health problem in the country.

The study also reveals that the spatial pattern of IPV was not random in Ethiopia. The Global Moran I value of 0.26 and the Z value of 8.29 with a *P* value < 0.0001 indicate that there has been a significant clustering of domestic violence in the country. This means that the distribution of IPV violence cases varies from one part of the country to the other. The Anselin Local hot spot analysis identifies hot spots, cold spots, and outlier clusters of IPV. Local Anselin hot spot analysis identifies hot spots, cold spots, and outlying clusters of IPV. Hotspot clusters are zones where high values are surrounded by high values and cold spots are zones where low values are surrounded by similar values. On the other hand, clusters are called outliers when high values are enclosed with low values or vice versa. In this study, hot spot clusters of IPV were observed in Amhara, Oromia, and SNNP regional states. Of the Amhara region, particularly in the East and West Gojam areas, the North and South Gondar areas, and the South Wollo area. In the Oromia region (West Arsi, Guji, Bale, and Jimma areas), and in the SNNP (Sidama, Gedio, Dawro, and Gamo Gofa areas) were the principals. In these regions, groups of women who have experienced IPV have been observed. This suggests that the magnitude of the problem is high and requires the attention of the responsible agencies.

The majority of respondents from the Amhara region reported that their husbands drunk alcohol. And, those from Oromia and SNNP regions accept wife-beating as justified action for the husband. This shows that Individual and community-level factors such as high level of alcohol consumption and community norm that encourage VAW may be responsible for spatial variation in the distribution of IPV. The spatial clustering of domestic violence cases was also reported from a study conducted in Brazil [13], a spatial epidemiologic study conducted in Spain [11], and from a study conducted in Rwanda [14].

The result from Sat Scan analysis of the data identifies primary and secondary most likely clusters. The primary significant cluster was located in Oromia (Guji and Borena zones), Somali (Liben and Afder zones), and SNNP (Sidama zone) regions of the country. The secondary cluster was located in the Amhara region east Gojam Zone and in Oromia in the Jimma zone. Women living in these clusters have a high risk of experiencing domestic violence when compared to those who reside outside these clusters. The identified clusters of IPV from Sat scan analysis were similar to the output from the anseline local cluster analysis indicating the identified locations of clusters were real. The results from previous studies conducted in foreign countries show that spatial variation in the distribution of intimate partner violence clusters was mainly attributed to neighborhood-level characteristics [9, 10, 15]. A high risk of intimate partner violence was observed among socio-economically disadvantaged communities, high immigrant concentration, and a high level of the public disorder [11]. In the current study, sufficient community-level variables (neighborhood level) were not included. Therefore, future studies may incorporate community-level predictors when conducting similar studies.

The result from logistic regression analysis shows that woman’s age is significantly associated with domestic violence. As a woman's age increases, the likelihood of experiencing domestic violence was also increased. The reason why older age women have a high risk of experiencing domestic violence when compared to younger ones may be because older women are more likely to be in a union for a longer time and this may increase their risk for violence. This result is consistent with an ecological study conducted in Brazil [16] and Nigeria [17].

The socio-economic status of women shows a significant association with domestic violence. Women from the richest family were 48% and those from richer 42% lower risk of domestic violence than those from the poorest households. This finding suggests that living in poverty plays a significant role in experiencing domestic violence. The finding from this study is supported by studies from Brazil [18], Zambia [4], Rwanda [14], and Ethiopia [8]. The relationship between low economic status and domestic violence may be explained by a partner with low income might not be able to support the household expense properly and, this might also be one cause for disputes.

This study also finds out that a partner’s alcohol use is significantly associated with experiencing domestic violence. A woman whose partner drink alcohol was 2.6 times more likely to experience domestic violence when compared to those whose partner doesn’t drink alcohol. This finding is in line with a previous study conducted on 14 sub-Saharan countries [19], with a study conducted in Ghana [2], Nigeria [17], Zambia [4], a systematic review in Ethiopia [8], and a study conducted in Southeast Ethiopia [20]. The result of this study is lower than a study conducted in southeast Oromia [21] and northwest Ethiopia [22]. This difference may be due to differences in the study population (because the previous studies are conducted mainly among pregnant women) and sample size differences (the current study was employed on large sample size). Despite this difference, harmful alcohol consumption by a partner is still considered the main risk factor for IPV. The main reason why women whose partners drink alcohol have a higher risk of domestic violence could be because excessive alcohol drinking may affect the cognitive function of the mind, reducing self-control and makes individuals incapable of a peaceful resolution to conflicts [23].

Respondent witnessing family violence as a child was also show significant association with experiencing domestic violence. A woman who saw family violence as childhood was 2.2 times more likely to experience domestic violence. This finding was consistent with a previous study conducted in Nigeria [17], southeast Oromia [21], and North West Ethiopia [24] but, lower than a study conducted in Ghana [2]. The difference in results between the current study and the study in Ghana may be due to population differences. The relationship between observing family violence and experience of domestic violence may be explained as a child who witnesses family violence may develop a behavioral or emotional problem in letter life and this could make him/her incapable to maintain stable relationship.

Marital controlling behaviors by partners also show significant association with experiencing domestic violence. Women whose partners exhibit at least one type of marital controlling behaviors were 4 times more likely to experience domestic violence when compared to those whose partners do not exhibit any type of marital controlling behaviors. The relationship between marital controlling behaviors and experience of domestic violence can be explained as: if one partner exhibits repetitive marital controlling behaviors, good feeling and effective communication will vanish between them and, this could lead to disputes and the occurrence of violence. The result of this study is in line with studies conducted in Brazil [25], Nigeria [26], and southwest Ethiopia [27].

Afraid of a partner was also strongly associated with experiencing domestic violence. Women who were occasionally afraid of their partners’ were 2 times and those who were frequently afraid were 6 times more likely to experience domestic violence when compared to those who do not afraid of their partners’. This finding is consistent with a study from Nepal [28] and Uganda [29]. Afraid of partner was considered as the result of many hostile behaviors and, it is mostly associated with many violent activities [29].

Social norms are community-level factors identified by previous studies to have a strong association with domestic violence. The analysis of social factors by different scholars shows that social norms can be manifested in two ways the first one is through gender norms and the second one is through gender norms perpetuating violence against women. Gender norms are informal social rules and expectations that distinguish males from females whereas gender norms perpetuating violence against women are norms that normalize violence within a specified community [30]. The current study focus on the second type of social norm; community acceptance of wife-beating that shows a strong association with a woman’s experience of domestic violence. Women who live in a community where wife-beating for husband is highly acceptable were 3.6 times more likely to experience domestic violence when compared to those who do not accept wife-beating. This result shows that the existence of a permissive social norm in the community plays a significant role in facing domestic violence by a woman. This finding is consistent with two previous studies from Nigeria [17, 31] and a study from Ethiopia [32].

This study has some limitations. The first one is sufficient community-level predictors of IPV were not included in this study. Therefore, it may not answer the reason why the distribution of IPV varies across communities properly. This may be one direction for future studies. The other limitation is to protect the confidentiality of respondents, location data in EDHS 2016 was displaced by 2 KM for urban areas and, 5 KM for rural. Thus, the study may not display the actual locations of IPV clusters.

## Conclusion

The output from the spatial analysis shows significant clustering of domestic violence cases in Ethiopia. Primary clusters were observed in southern Oromia, Somali, and some parts of SNNP whereas, secondary clusters were observed in Amhara and Oromia regional states.

In this study, a strong association of domestic violence with an individual, relationship, and community-level predictors were observed. The output from logistic regression shows that partner’s alcohol use, witnessing family violence as a child, marital controlling behaviors, fear of partner, and community acceptance of wife-beating were predictors of domestic violence.

Prevention and control of IPV is the shared responsibility of everyone. All parties including governmental organizations, non-government organizations, the scientific community, community leaders, and every individual should be involved. Based upon the findings, we forward the following recommendations:

- The government should give prior concern for controlling factors such as high level of alcohol consumption and social norms that encourage violence against women that were responsible for experiencing IPV in the identified hot spot areas when employing any interventional activities.
- Training on how to maintain stable relationships should be given to couples before getting into marriage.
- The scientific community needs to expose the hidden reality behind closed doors by conducting scientific researches.

## Data Availability

The datasets used for analysis was obtained from https://dhsprogram.com/Data/.

## Abbreviations

AIC: Akaike’s information criterion
AOR: Adjusted Odds ratio
BIC: Bayesian information criterion
CI: Confidence interval
CSA: Central statistical agency
DHS: Demographic and health survey
EAs: Enumeration Areas
EDHS: Ethiopian demographic health survey
ICC: Intracluster correlation coefficient
IPV: Intimate partner violence
LLR: Log-likelihood ratio
RR: Relative risk
SNNP: Southern Nations Nationalities and Peoples
USA: United States of America
VAW: Violence against women
WHO: World health organization
